# Trunk asymmetry in juveniles

**DOI:** 10.1186/1748-7161-3-13

**Published:** 2008-09-23

**Authors:** Theodoros B Grivas, Elias S Vasiliadis, Constantinos Mihas, Georgios Triantafyllopoulos, Angelos Kaspiris

**Affiliations:** 1 Orthopaedic Department, "Thriasio" General Hospital, G. Gennimata Av. 19600, Magoula, Attica, Greece

## Abstract

**Background:**

Trunk asymmetry (TA) is a common phenomenon in children, but its incidence in juveniles is not known. The present cross sectional study reports TA in normal juveniles and provides data which describe the evolution of TA from early childhood to adolescence.

**Materials and methods:**

The scoliometer readings in both standing and sitting forward bending position (FBP) of 3301 children, (1645 boys, and 1656 girls) aged from 3 to 9 years old were studied. TA was quantified by measuring angle of trunk rotation (ATR) and children were categorized as symmetric (ATR = 0°), mild asymmetric (ATR 1° – 6°) and severely asymmetric (ATR ≥ 7°). The difference of TA between standing and sitting FBP as well as differences between boys and girls in frequency of TA were also calculated. The scoliometer readings were analyzed by age to reveal at which age the juvenile pattern of TA changes into the adolescent one.

**Results:**

74.2% of boys and 77% of girls were symmetric (ATR = 0°) in the thoracic region in standing FBP, while 82.7% of boys and 84.1% of girls were symmetric in the thoracic region in sitting FBP. Juvenile girls are more symmetric than boys but severe TA was found almost the same between the two genders. A significant reduction in the frequency of mild TA from standing into sitting FBP, in all the examined regions in both boys and girls was found, but in severe TA this reduction is very small. Analysing scoliometer readings by age it appears that significant TA changes take place between 8–9 years of age for boys and between 6–7 and 8–9 years for girls. TA in boys is changing into the adolescent pattern at a later age than in girls.

**Conclusion:**

Juveniles were found more symmetric than adolescents, who were studied previously in a different study. Furthermore, juvenile girls were found more symmetric than boys. Juvenile TA pattern seems to be in accordance with the higher incidence of juvenile idiopathic scoliosis in boys. Furthermore, severe TA, which could be correlated with a scoliotic curve, was found to be more common to the left side. The present report provides information about the variability of back morphology in normal juveniles. The amount of TA in children is the indicator for referral and further orthopaedic assessment if a spinal curve is detected, but can also be used as a baseline for further research on idiopathic scoliosis aetiology.

## Background

Trunk asymmetry (TA) is a relatively common phenomenon [1-5] and was found to correlate well with the prediction of future scoliosis in adolescents [6]. In a previous study we reported that TA is not a sensitive clinical sign for scoliosis in juvenile girls who were referred from a school-screening program [7]. In the same study, 25% of children with Angle of Trunk Rotation (ATR) ≥ 7° were found to have either a straight spine or a curve smaller than 10° [7,8].

TA appears early in childhood and does not correlate with spinal deformity until adolescence [7]. This observation clearly demonstrates the effect of growth in the pathogenesis of idiopathic scoliosis.

Since the incidence of TA in juveniles is not known, we conducted the present cross sectional study to quantify for the first time TA in normal juveniles and to provide data which describe the evolution of TA from early childhood to adolescence.

## Methods

### The measurements

The scoliometer (Pruijs scoliometer, Orthomet-Surgeyplant B.V. Waalwijl, Netherlands), readings in both standing and sitting forward bending position (FBP) of 3301 children, (1645 boys, and 1656 girls) aged from 3 to 9 years old were studied. The children who were included in the study were examined during school screening program, between 1996 and 2006. Children younger than 5 years of age were examined at the scoliosis clinic of our department. TA was quantified by measuring angle of trunk rotation (ATR) at mid-thoracic (T4-T8), thoraco-lumbar (T12-L1) and at the lumbar (L2-L5) regions of the back. The child was asked to bend forward, looking down, keeping the feet 15 cm apart, knees braced back, shoulders loose and hands positioned in front of knees or shins with elbows straight and palms opposed. Any leg length inequality was not corrected. The side of the hump determined the laterality of trunk rotation. Trunk asymmetry to the right side (higher hump on the right) was defined as right asymmetry and to the left (higher hump on the left) was defined as left asymmetry in each of the three mentioned regions and recorded in degrees.

In the sitting forward bending position, the student was seated on a chair (40 cm high) and was asked to bend forwards and place the head between the knees with the shoulders loose, elbows straight and hands positioned between knees. The scoliometer measurements were obtained successively at the same three areas of interest as in the standing forward bending position.

The reliability study of the scoliometer measurements has been analyzed and reported previously [9].

### The examined children

Children were categorized as symmetric (ATR = 0°), mild asymmetric (ATR 1° – 6°) and severely asymmetric (ATR ≥ 7°). Frequency of symmetry, mild and severe TA for both boys and girls was quantified in standing and in sitting FBP for all the examined regions of the back. The difference of TA between standing and sitting FBP as well as differences between boys and girls in frequency of TA were also calculated. The scoliometer readings were analyzed by age to reveal at which age the juvenile pattern of TA changes into the adolescent one.

## Results

**Figure 1:**
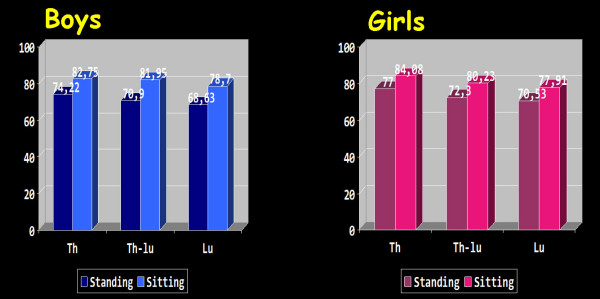
**Frequency of symmetry (%) in the three examined regions of the spine in boys and girls in both standing and sitting position.** Th: Thoracic region, Th-Lu: thoracolumbar region, Lu: lumbar region.

**Figure 2:**
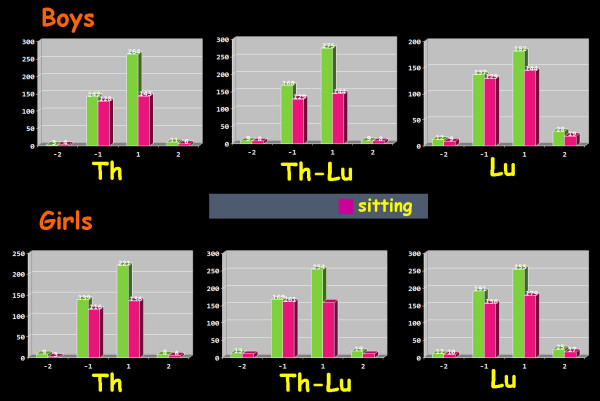
**Number of asymmetric boys and girls in the three examined regions of the spine in both standing and sitting position. **1: mild right asymmetry (ATR 1–6°), 2: severe right asymmetry (ATR ≥ 7°), -1 mild left asymmetry (ATR 1–6°), -2: severe left asymmetry (ATR ≥ 7°). Th: Thoracic region, Th-Lu: thoracolumbar region, Lu: lumbar region.

Seventy four per cent of boys and seventy seven per cent of girls were symmetric (ATR = 0°) in the thoracic region in standing FBP, while 82.7% of boys and 84.1% of girls were symmetric in the thoracic region in sitting FBP. For the thoracolumbar region, 70.9% of boys and 72.3% of girls were symmetric (ATR = 0°) in standing FBP, while 82% of boys and 80.2% of girls were symmetric in the thoracolumbar region in sitting FBP. For lumbar region 68.6% of boys and 70.5% of girls were symmetric (ATR = 0°) in standing FBP, while 78.7% of boys and 77.9% of girls were symmetric in sitting FBP, [Tables 1, 2, 3 and 4, Figures 1 and 2].

**Table 1:** Frequency of symmetry and asymmetry in *boys*.

		**Symmetry**	**1**	**-1**	**2**	**-2**	**Missing**	**Total**
**Thoracic**	N	**1209**	**264**	**142**	**11**	**3**	**16**	**1645**
	%	*74.22*	*16.21*	*8.72*	*0.68*	*0.18*		*100.00*
**Thoraco – lumbar**	N	**1155**	**275**	**168**	**22**	**9**	**16**	**1645**
	%	*70.90*	*16.88*	*10.31*	*1.35*	*0.55*		*100.00*
**Lumbar**	N	**1118**	**302**	**169**	**28**	**12**	**16**	**1645**
	%	*68.63*	*18.54*	*10.37*	*1.72*	*0.74*		*100.00*

Scoliometer readings are obtained at *standing *forward bending position. 1: mild right asymmetry (ATR 1–6°), 2: severe right asymmetry (ATR ≥ 7°), -1 mild left asymmetry (ATR 1–6°), -2: severe left asymmetry (ATR ≥ 7°).

**Table 2:** Frequency of symmetry and asymmetry in *girls*.

		**Symmetry**	**1**	**-1**	**2**	**-2**	**Missing**	**Total**
**Thoracic**	N	**1262**	**221**	**139**	**8**	**9**	**17**	**1656**
	%	*77.00*	*13.48*	*8.48*	*0.49*	*0.55*		*100.00*
**Thoraco – lumbar**	N	**1185**	**254**	**168**	**19**	**13**	**17**	**1656**
	%	*72.30*	*15.50*	*10.25*	*1.16*	*0.79*		*100.00*
**Lumbar**	N	**1156**	**255**	**191**	**25**	**12**	**17**	**1656**
	%	*70.53*	*15.56*	*11.65*	*1.53*	*0.73*		*100.00*

Scoliometer readings are obtained at *standing *forward bending position. 1: mild right asymmetry (ATR 1–6°), 2: severe right asymmetry (ATR ≥ 7°), -1 mild left asymmetry (ATR 1–6°), -2: severe left asymmetry (ATR ≥ 7°).

**Table 3:** Frequency of symmetry and asymmetry in *boys*.

		**Symmetry**	**1**	**-1**	**2**	**-2**	**Missing**	**Total**
**Thoracic**	N	**1348**	**143**	**128**	**6**	**4**	**16**	**1645**
	%	*82.75*	*8.78*	*7.86*	*0.37*	*0.25*		*100.00*
**Thoraco – lumbar**	N	**1335**	**144**	**129**	**13**	**8**	**16**	**1645**
	%	*81.95*	*8.84*	*7.92*	*0.80*	*0.49*		*100.00*
**Lumbar**	N	**1282**	**182**	**137**	**19**	**9**	**16**	**1645**
	%	*78.70*	*11.17*	*8.41*	*1.17*	*0.55*		100

Scoliometer readings are obtained at *sitting *forward bending position. 1: mild right asymmetry (ATR 1–6°), 2: severe right asymmetry (ATR ≥ 7°), -1 mild left asymmetry (ATR 1–6°), -2: severe left asymmetry (ATR ≥ 7°).

**Table 4:** Frequency of symmetry and asymmetry in *girls*.

		**Symmetry**	**1**	**-1**	**2**	**-2**	**Missing**	**Total**
**Thoracic**	N	**1378**	**136**	**116**	**6**	**3**	**17**	**1656**
	%	*84.08*	*8.30*	*7.08*	*0.37*	*0.18*		*100.00*
**Thoraco – lumbar**	N	**1315**	**159**	**141**	**12**	**12**	**17**	**1656**
	%	*80.23*	*9.70*	*8.60*	*0.73*	*0.73*		*100.00*
**Lumbar**	N	**1277**	**179**	**156**	**17**	**10**	**17**	**1656**
	%	*77.91*	*10.92*	*9.52*	*1.04*	*0.61*		*100.00*

Scoliometer readings are obtained at *sitting *forward bending position. 1: mild right asymmetry (ATR 1–6°), 2: severe right asymmetry (ATR ≥ 7°), -1 mild left asymmetry (ATR 1–6°), -2: severe left asymmetry (ATR ≥ 7°).

Mild asymmetry (ATR 1° – 6°) in the thoracic region was found in 24.9% of boys and in 22% of girls in standing FBP and in 15.6% of boys and in 15.4% of girls in sitting FBP. In the thoracolumbar region mild asymmetry was found in 27.2% of boys and in 25.8% of girls in standing FBP and in 16.8% of boys and in 18.3% of girls in sitting FBP. In lumbar region mild asymmetry was found in 28.9% of boys and in 27.2% of girls in standing FBP and in 19.6% of boys and in 20.4% of girls in sitting FBP, [Tables 1, 2, 3, 4, Figures 1, 2].

Severe asymmetry (ATR 1° – 6°) in the thoracic region was found in 0.9% of boys and in 1% of girls in standing FBP and in 0.6% of boys and in 0.6% of girls in sitting FBP. In the thoracolumbar region severe asymmetry was found in 1.9% of boys and in 2% of girls in standing FBP and in 1.3% of boys and in 1.5% of girls in sitting FBP. In lumbar region severe asymmetry was found in 2.5% of boys and in 2.3% of girls in standing FBP and in 1.7% of boys and in 1.7% of girls in sitting FBP, [Tables 1, 2, 3, 4, Figures 1, 2].

The distribution of mild and severe TA (right or left) in standing and sitting FBP in the three examined regions of the spine in both boys and girls is shown in Figure 2.

**Figure 3:**
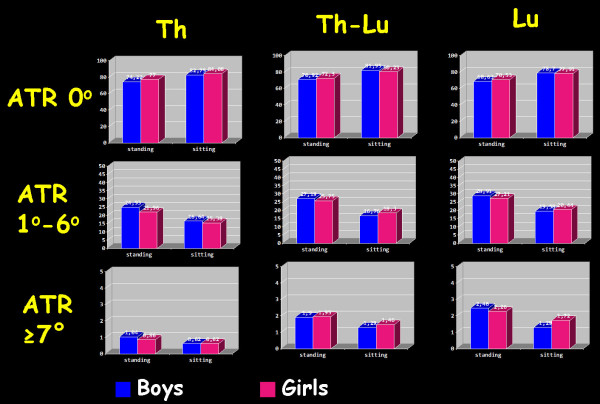
**Boys/Girls difference of symmetry, mild and severe asymmetry in the three examined regions of the spine in both standing and sitting position.** Th: Thoracic region, Th-Lu: thoracolumbar region, Lu: lumbar region. ATR: Angle of Trunk Rotation.

Girls were found more symmetric than boys but severe TA was found almost the same between the two genders, [Figure 3].

A significant reduction in the frequency of mild TA from standing into sitting FBP, in all the examined regions in both boys and girls was found, [Table 5]. This difference in the thoracic region is 8.5% in boys and 7.1% in girls for symmetric children, while in the thoracolumbar region is 11% in boys and 7.9% in girls and in the lumbar region is 10.1% in boys and 7.4% in girls. In severely asymmetric children the difference is very small, and is measured 0.4%, 0.6% and 0.7% in boys in the thoracic, thoracolumbar and lumbar region respectively and 0.5%, 0.5% and 0.6% in girls in the thoracic, thoracolumbar and lumbar region respectively, [Table 5].

**Table 5:** The difference (%) of the frequency of asymmetry between standing and sitting FBP, in the three examined regions of the spine, for both boys and girls.

	**Boys**			**Girls**		
	**Symmetry ATR = 0°**	**ATR 1°–6°**	**ATR ≥ 7°**	**Symmetry ATR = 0°**	**ATR 1°–6°**	**ATR ≥ 7°**
**Thoracic**	8,5%	8,3%	0,4%	7,1%	6,6%	0,5%
**Thoracolumbar**	11%	10,4%	0,6%	7,9%	7,5%	0,5%
**Lumbar**	10,1%	9,3%	0,7%	7,4%	6,8%	0,6%

Analysing scoliometer readings by age [Tables 6, 7, 8 and 9] it appears that significant TA changes take place between 8–9 years of age for boys and between 6–7 and 8–9 years for girls. TA in boys is changing into the adolescent pattern at a later age than in girls.

**Table 6:** Frequency of symmetry and asymmetry in *boys by age (year)*.

	**Thoracic**												
		**Symmetry**		**1**		**-1**		**2**		**-2**		**Total**	
		**N**	*%*	**N**	*%*	**N**	*%*	**N**	*%*	**N**	*%*	**N**	*%*
Age	3	**3**	*0.25*									**3**	*0.18*
	4	**4**	*0.33*									**4**	*0.25*
	5	**22**	*1.82*	**1**	*0.38*	**2**	*1.41*					**25**	*1.53*
	6	**231**	*19.11*	**55**	*20.83*	**21**	*14.79*	**1**	*9.09*	**2**	*66.67*	**310**	*19.03*
	7	**311**	*25.72*	**56**	*21.21*	**37**	*26.06*	**5**	*45.45*			**409**	*25.11*
	8	**297**	*24.57*	**68**	*25.76*	**29**	*20.42*	**1**	*9.09*	**1**	*33.33*	**396**	*24.31*
	9	**341**	*28.21*	**84**	*31.82*	**53**	*37.32*	**4**	*36.36*			**482**	*29.59*
Total		**1209**	*100.00*	**264**	*100.00*	**142**	*100.00*	**11**	*100.00*	**3**	*100.00*	**1629**	*100.00*
	**Thoracolumbar**												
		**Symmetry**		**1**		**-1**		**2**		**-2**		**Total**	
		**N**	*%*	**N**	*%*	**N**	*%*	**N**	*%*	**N**	*%*	**N**	*%*
Age	3	**2**	*0.17*	**1**	*0.36*							**3**	*0.18*
	4	**3**	*0.26*			**1**	*0.60*					**4**	*0.25*
	5	**21**	*1.82*	**3**	*1.09*	**1**	*0.60*					**25**	*1.53*
	6	**230**	*19.91*	**50**	*18.18*	**28**	*16.67*	**1**	*4.55*	**1**	*11.11*	**310**	*19.03*
	7	**295**	*25.54*	**57**	*20.73*	**43**	*25.60*	**9**	*40.91*	**5**	*55.56*	**409**	*25.11*
	8	**273**	*23.64*	**75**	*27.27*	**42**	*25.00*	**5**	*22.73*	**1**	*11.11*	**396**	*24.31*
	9	**331**	*28.66*	**89**	*32.36*	**53**	*31.55*	**7**	*31.82*	**2**	*22.22*	**482**	*29.59*
Total		**1155**	*100.00*	**275**	*100.00*	**168**	*100.00*	**22**	*100.00*	**9**	*100.00*	**1629**	*100.00*
	**Lumbar**												
		**Symmetry**		**1**		**-1**		**2**		**-2**		**Total**	
		**N**	*%*	**N**	*%*	**N**	*%*	**N**	*%*	**N**	*%*	**N**	*%*
Age	3	**2**	*0.18*	**1**	*0.33*							**3**	*0.18*
	4	**4**	*0.36*									**4**	*0.25*
	5	**17**	*1.52*	**5**	*1.66*	**2**	*1.18*	**1**	*3.57*			**25**	*1.53*
	6	**223**	*19.95*	**62**	*20.53*	**21**	*12.43*	**2**	*7.14*	**2**	*16.67*	**310**	*19.03*
	7	**270**	*24.15*	**81**	*26.82*	**43**	*25.44*	**9**	*32.14*	**6**	*50.00*	**409**	*25.11*
	8	**274**	*24.51*	**72**	*23.84*	**43**	*25.44*	**6**	*21.43*	**1**	*8.33*	**396**	*24.31*
	9	**328**	*29.34*	**81**	*26.82*	**60**	*35.50*	**10**	*35.71*	**3**	*25.00*	**482**	*29.59*
Total		**1118**	*100.00*	**302**	*100.00*	**169**	*100.00*	**28**	*100.00*	**12**	*100.00*	**1629**	*100.00*

Scoliometer readings are at *standing *forward bending position. 1: mild right asymmetry (ATR 1–6o), 2: severe right asymmetry (ATR ≥ 7o), -1 mild left asymmetry (ATR 1–6o), -2: severe left asymmetry (ATR ≥ 7o).

**Table 7:** Frequency of symmetry and asymmetry in *boys by age (year)*.

	**Thoracic**												
		**Symmetry**		**1**		**-1**		**2**		**-2**		**Total**	
		**N**	*%*	**N**	*%*	**N**	*%*	**N**	*%*	**N**	*%*	**N**	*%*
Age	3	**3**	*0.22*									**3**	*0.18*
	4	**4**	*0.30*									**4**	*0.25*
	5	**24**	*1.78*	**1**	*0.70*							**25**	*1.53*
	6	**256**	*18.99*	**24**	*16.78*	**28**	*21.88*	**2**	*33.33*			**310**	*19.03*
	7	**336**	*24.93*	**42**	*29.37*	**30**	*23.44*	**1**	*16.67*			**409**	*25.11*
	8	**329**	*24.41*	**34**	*23.78*	**30**	*23.44*			**3**	*75.00*	**396**	*24.31*
	9	**396**	*29.38*	**42**	*29.37*	**40**	*31.25*	**3**	*50.00*	**1**	*25.00*	**482**	*29.59*
Total		**1348**	*100.00*	**143**	*100.00*	**128**	*100.00*	**6**	*100.00*	**4**	*100.00*	**1629**	*100.00*
	**Thoracolumbar**												
		**Symmetry**		**1**		**-1**		**2**		**-2**		**Total**	
		**N**	*%*	**N**	*%*	**N**	*%*	**N**	*%*	**N**	*%*	**N**	*%*
Age	3	**2**	*0.15*	**1**	*0.69*							**3**	*0.18*
	4	**4**	*0.30*									**4**	*0.25*
	5	**24**	*1.80*	**1**	*0.69*							**25**	*1.53*
	6	**257**	*19.25*	**21**	*14.58*	**30**	*23.26*	**1**	*7.69*	**1**	*12.50*	**310**	*19.03*
	7	**328**	*24.57*	**41**	*28.47*	**34**	*26.36*	**3**	*23.08*	**3**	*37.50*	**409**	*25.11*
	8	**321**	*24.04*	**36**	*25.00*	**33**	*25.58*	**3**	*23.08*	**3**	*37.50*	**396**	*24.31*
	9	**399**	*29.89*	**44**	*30.56*	**32**	*24.81*	**6**	*46.15*	**1**	*12.50*	**482**	*29.59*
Total		**1335**	*100.00*	**144**	*100.00*	**129**	*100.00*	**13**	*100.00*	**8**	*100.00*	**1629**	*100.00*
	**Lumbar**												
		**Symmetry**		**1**		**-1**		**2**		**-2**		**Total**	
		**N**	*%*	**N**	*%*	**N**	*%*	**N**	*%*	**N**	*%*	**N**	*%*
Age	3	**2**	*0.16*	**1**	*0.55*							**3**	*0.18*
	4	**4**	*0.31*									**4**	*0.25*
	5	**22**	*1.72*	**1**	*0.55*	**1**	*0.73*	**1**	*5.26*			**25**	*1.53*
	6	**247**	*19.27*	**36**	*19.78*	**21**	*15.33*	**3**	*15.79*	**3**	*33.33*	**310**	*19.03*
	7	**316**	*24.65*	**49**	*26.92*	**35**	*25.55*	**7**	*36.84*	**2**	*22.22*	**409**	*25.11*
	8	**310**	*24.18*	**44**	*24.18*	**34**	*24.82*	**5**	*26.32*	**3**	*33.33*	**396**	*24.31*
	9	**381**	*29.72*	**51**	*28.02*	**46**	*33.58*	**3**	*15.79*	**1**	*11.11*	**482**	*29.59*
Total		**1282**	*100.00*	**182**	*100.00*	**137**	*100.00*	**19**	*100.00*	**9**	*100.00*	**1629**	*100.00*

Scoliometer readings are at *sitting *forward bending position. 1: mild right asymmetry (ATR 1–6o), 2: severe right asymmetry (ATR ≥ 7o), -1 mild left asymmetry (ATR 1–6o), -2: severe left asymmetry (ATR ≥ 7o).

**Table 8:** Frequency of symmetry and asymmetry in *girls by age (year)*.

	**Thoracic**												
		**Symmetry**		**1**		**-1**		**2**		**-2**		**Total**	
		**N**	*%*	**N**	*%*	**N**	*%*	**N**	*%*	**N**	*%*	**N**	*%*
Age	3	**3**	*0.24*			**1**	*0.72*					**4**	*0.24*
	4	**4**	*0.40*	**1**	*0.45*							**6**	*0.37*
	5	**22**	*2.61*	**2**	*0.90*							**35**	*2.14*
	6	**231**	*21.39*	**41**	*18.55*	**23**	*16.55*	**1**	*12.50*	**2**	*22.22*	**337**	*20.56*
	7	**311**	*22.35*	**48**	*21.72*	**33**	*23.74*	**3**	*37.50*	**2**	*22.22*	**368**	*22.45*
	8	**297**	*29.48*	**59**	*26.70*	**35**	*25.18*	**3**	*37.50*	**3**	*33.33*	**472**	*28.80*
	9	**341**	*23.53*	**70**	*31.67*	**47**	*33.81*	**1**	*12.50*	**2**	*22.22*	**417**	*25.44*
Total		**1209**	*100.00*	**221**	*100.00*	**139**	*100.00*	**8**	*100.00*	**9**	*100.00*	**1639**	*100.00*
	**Thoracolumbar**												
		**Symmetry**		**1**		**-1**		**2**		**-2**		**Total**	
		**N**	*%*	**N**	*%*	**N**	*%*	**N**	*%*	**N**	*%*	**N**	*%*
Age	3	**2**	*0.25*			**1**	*0.60*					**4**	*0.24*
	4	**3**	*0.42*	**1**	*0.39*							**6**	*0.37*
	5	**21**	*2.62*	**4**	*1.57*							**35**	*2.14*
	6	**230**	*21.01*	**54**	*21.26*	**27**	*16.07*	**4**	*21.05*	**3**	*23.08*	**337**	*20.56*
	7	**295**	*22.45*	**54**	*21.26*	**40**	*23.81*	**4**	*21.05*	**4**	*30.77*	**368**	*22.45*
	8	**273**	*28.95*	**67**	*26.38*	**54**	*32.14*	**6**	*31.58*	**2**	*15.38*	**472**	*28.80*
	9	**331**	*24.30*	**74**	*29.13*	**46**	*27.38*	**5**	*26.32*	**4**	*30.77*	**417**	*25.44*
Total		**1155**	*100.00*	**254**	*100.00*	**168**	*100.00*	**19**	*100.00*	**13**	*100.00*	**1639**	*100.00*
	**Lumbar**												
		**Symmetry**		**1**		**-1**		**2**		**-2**		**Total**	
		**N**	*%*	**N**	*%*	**N**	*%*	**N**	*%*	**N**	*%*	**N**	*%*
Age	3	**2**	*0.26*			**1**	*0.52*					**4**	*0.24*
	4	**4**	*0.35*	**2**	*0.78*							**6**	*0.37*
	5	**17**	*2.85*	**2**	*0.78*							**35**	*2.14*
	6	**223**	*20.50*	**59**	*23.14*	**35**	*18.32*	**3**	*12.00*	**3**	*25.00*	**337**	*20.56*
	7	**270**	*22.32*	**57**	*22.35*	**37**	*19.37*	**10**	*40.00*	**6**	*50.00*	**368**	*22.45*
	8	**274**	*29.76*	**61**	*23.92*	**62**	*32.46*	**4**	*16.00*	**1**	*8.33*	**472**	*28.80*
	9	**328**	*23.96*	**74**	*29.02*	**56**	*29.32*	**8**	*32.00*	**2**	*16.67*	**417**	*25.44*
Total		**1118**	*100.00*	**255**	*100.00*	**191**	*100.00*	**25**	*100.00*	**12**	*100.00*	**1639**	*100.00*

Scoliometer readings are at *standing *forward bending position. 1: mild right asymmetry (ATR 1–6o), 2: severe right asymmetry (ATR ≥ 7o), -1 mild left asymmetry (ATR 1–6o), -2: severe left asymmetry (ATR ≥ 7o).

**Table 9:** Frequency of symmetry and asymmetry in *girls by age (year)*.

	**Thoracic**												
		**Symmetry**		**1**		**-1**		**2**		**-2**		**Total**	
		**N**	*%*	**N**	*%*	**N**	*%*	**N**	*%*	**N**	*%*	**N**	*%*
Age	3	**3**	*0.29*									**4**	*0.24*
	4	**4**	*0.29*	**2**	*1.47*							**6**	*0.37*
	5	**24**	*2.39*	**2**	*1.47*							**35**	*2.14*
	6	**256**	*21.04*	**30**	*22.06*	**16**	*13.79*	**1**	*16.67*			**337**	*20.56*
	7	**336**	*22.06*	**28**	*20.59*	**33**	*28.45*	**3**	*50.00*			**368**	*22.45*
	8	**329**	*29.39*	**33**	*24.26*	**32**	*27.59*	**2**	*33.33*			**472**	*28.80*
	9	**396**	*24.53*	**41**	*30.15*	**35**	*30.17*			**3**	*100.00*	**417**	*25.44*
Total		**1348**	*100.00*	**136**	*100.00*	**116**	*100.00*	**6**	*100.00*	**3**	*100.00*	**1639**	*100.00*
	**Thoracolumbar**												
		**Symmetry**		**1**		**-1**		**2**		**-2**		**Total**	
		**N**	*%*	**N**	*%*	**N**	*%*	**N**	*%*	**N**	*%*	**N**	*%*
Age	3	**2**	*0.30*									**4**	*0.24*
	4	**4**	*0.30*	**2**	*1.26*							**6**	*0.37*
	5	**24**	*2.43*	**2**	*1.26*	**1**	*0.71*					**35**	*2.14*
	6	**257**	*21.67*	**31**	*19.50*	**17**	*12.06*	**2**	*16.67*	**2**	*16.67*	**337**	*20.56*
	7	**328**	*22.66*	**31**	*19.50*	**30**	*21.28*	**5**	*41.67*	**4**	*33.33*	**368**	*22.45*
	8	**321**	*28.75*	**42**	*26.42*	**47**	*33.33*	**3**	*25.00*	**2**	*16.67*	**472**	*28.80*
	9	**399**	*23.88*	**51**	*32.08*	**46**	*32.62*	**2**	*16.67*	**4**	*33.33*	**417**	*25.44*
Total		**1335**	*100.00*	**159**	*100.00*	**141**	*100.00*	**12**	*100.00*	**12**	*100.00*	**1639**	*100.00*
	**Lumbar**												
		**Symmetry**		**1**		**-1**		**2**		**-2**		**Total**	
		**N**	*%*	**N**	*%*	**N**	*%*	**N**	*%*	**N**	*%*	**N**	*%*
Age	3	**2**	*0.31*									**4**	*0.24*
	4	**4**	*0.31*	**1**	*0.56*			**1**	*5.88*			**6**	*0.37*
	5	**22**	*2.43*	**4**	*2.23*							**35**	*2.14*
	6	**247**	*21.14*	**38**	*21.23*	**27**	*17.31*	**1**	*5.88*	**1**	*10.00*	**337**	*20.56*
	7	**316**	*21.85*	**39**	*21.79*	**40**	*25.64*	**6**	*35.29*	**4**	*40.00*	**368**	*22.45*
	8	**310**	*29.37*	**42**	*23.46*	**45**	*28.85*	**6**	*35.29*	**4**	*40.00*	**472**	*28.80*
	9	**381**	*24.59*	**55**	*30.73*	**44**	*28.21*	**3**	*17.65*	**1**	*10.00*	**417**	*25.44*
Total		**1282**	*100.00*	**179**	*100.00*	**156**	*100.00*	**17**	*100.00*	**10**	*100.00*	**1639**	*100.00*

Scoliometer readings are at *sitting *forward bending position. 1: mild right asymmetry (ATR 1–6o), 2: severe right asymmetry (ATR ≥ 7o), -1 mild left asymmetry (ATR 1–6o), -2: severe left asymmetry (ATR ≥ 7o).

## Discussion

In the present study the incidence of TA was greater in the standing than in the sitting FBP, both for boys and girls, in all the examined regions of the spine. The same was found in adolescents [4], which imply leg length or pelvic difference in pathogenesis of TA. Leg length or pelvic difference may force the trunk to rotate so that the body can maintain its balance. In healthy children a physiological shortening of one leg is associated with a contralateral hump on the back in forward flexion. Small differences in the lengths of the lower limbs would have a mild effect on the shape of the surface of the back in the lumbar region, and a minimum effect in the thoracic region in forward bending position [10]. Furthermore, when a child with normal lumbar lordosis flexes forward above a tilted pelvis, a small loin hump is produced because the lumbar spine rotates to avoid compression. [11].

In the studied population we found that severe TA, which is associated with a possible scoliotic curve, is more common to the left side. The main factor among others is the usually longer right leg in children prior to puberty [12], a pattern which changes with growth. Considering that most right-handed children preferentially use the left leg, a greater working load is imposed on it, inducing growth acceleration and resulting in a longer left leg at a later age [12] and thus a possible change in the TA pattern. Leg asymmetry in normal children is either equalized during growth or with the contribution of other mechanisms, facilitate the increase of TA and probably is involved in the pathogenesis of idiopathic scoliosis [13]. The typical asymmetric pelvis has also its left half set a little higher and further back than the right [12,14]. The examination of TA, with the child placed in sitting FBP expresses the true TA, which is revealed due to the levelling of the pelvis and elimination of any effect of leg length difference on back shape.

Juveniles were found more symmetric than adolescents. Seventy four per cent of juvenile boys and seventy seven per cent of juvenile girls were found symmetric in the thoracic region of the spine in the standing FBP, which is significantly higher than the sixty eight per cent of adolescent boys and sixty five per cent of adolescent girls, who were found symmetric in a previous study [4]. Similar differences exist in TA when examining the child in sitting FBP.

In summary, TA in juveniles (juvenile pattern), which is found to be less frequent than adolescents, is more common in boys and to the left side, while in adolescents (adolescent pattern), TA is more common in girls and to the right side [4].

Growth is more than an increase in size and involves maturational processes in the child [15]. In normal health the skeletal system and nervous system evidently grow and mature together [16]. The dynamic physiological balance of body shape is continuously renewed between two synchronous processes *(escalators) *[16], namely: 1) *increasing skeletal size and relative segmental mass*, and 2) the *Central Nervous System (CNS) body schema*. Such normal neuro-osseous development occurs in the limbs, spine and trunk [17,18]. When comparing TA between girls and boys, juvenile girls were found more symmetric than juvenile boys, a finding which is opposite in adolescents [4,9]. Juvenile TA pattern seems to be in accordance with the higher incidence of juvenile idiopathic scoliosis in boys [19,20]. Furthermore, severe TA, which implies the existence of a scoliotic curve, was found to be more common to the left side. There must be a transitional age, when growth affects the child's back and results into changes of TA from juvenile into an adolescent pattern. These changes seem to be more important in girls. Girls, by entering their adolescent skeletal growth spurt in postural immaturity, compared with boys who enter their adolescent growth spurt in postural maturity, may disturb the neuro-osseous balance of the 'escalators', resulting in an increased incidence of TA at an older age. Growth is gradually completed in three directions, namely dorsoventral, craniocaudal, and clockwise (left-right), which is a rather symmetric process and is the expression of control genes [21]. Any of the above mentioned directional elements of symmetry is lost when the developmental program coded in the genome fails to run optimally. This failure can be attributed to certain stress factors (intrinsically genetic or environmental) [21]. The dominance of left severe asymmetry in juveniles as it is documented in this study is in accordance to the above developmental theory, and especially the clockwise (left-right) element, where left is pronounced in younger and right asymmetries in older ages. Furthermore, increased incidence of TA in older girls is itself a destabilizing influence and can account for the increased incidence of spinal deformity in female patients.

In the present study we found that TA in boys is changing into the adolescent pattern at a later age than in girls. This observation confirms that girls reach maturity earlier than boys and is in accordance with the existing reports in the literature [22].

The amount of TA in children is the indicator for referral and further orthopaedic assessment if a spinal curve is detected, but can also be used as a baseline for further research on idiopathic scoliosis aetiology. The present report provides information about the variability of back morphology in normal juveniles which is worth knowing when a child is examined for juvenile scoliosis.

## Abbreviations

TA: Trunk asymmetry; FBP: Forward Bending Position; ATR: Angle of Trunk Rotation; CNS: Central Nervous System.

## Authors' contributions

TBG conceived the idea of the presented study, performed part of the literature review, contributed to the interpretation of data and in the drafting of the manuscript. EV performed part of the literature review, contributed in the interpretation of data and in manuscript drafting. CM contributed in the interpretation of data. GT and AK performed part of the literature review and participated in the school screening program. All authors have read and approved the final manuscript.
